# Sub-Chronic Microcystin-LR Liver Toxicity in Preexisting Diet-Induced Nonalcoholic Steatohepatitis in Rats

**DOI:** 10.3390/toxins11070398

**Published:** 2019-07-09

**Authors:** Tarana Arman, Katherine D. Lynch, Michelle L. Montonye, Michael Goedken, John D. Clarke

**Affiliations:** 1 Department of Pharmaceutical Sciences, Washington State University, Spokane, WA 99202, USA; 2 Department of Pharmacology and Toxicology, Rutgers University, Piscataway, NJ 08901, USA

**Keywords:** microcystin-LR, nonalcoholic steatohepatitis, sub-chronic toxicity

## Abstract

Microcystin-LR (MCLR) is a hepatotoxic cyanotoxin reported to cause a phenotype similar to nonalcoholic steatohepatitis (NASH). NASH is a common progressive liver disease that advances in severity due to exogenous stressors such as poor diet and toxicant exposure. Our objective was to determine how sub-chronic MCLR toxicity affects preexisting diet-induced NASH. Sprague-Dawley rats were fed one of three diets for 10 weeks: control, methionine and choline deficient (MCD), or high fat/high cholesterol (HFHC). After six weeks of diet, animals received vehicle, 10 µg/kg, or 30 µg/kg MCLR via intraperitoneal injection every other day for the final 4 weeks. Incidence and severity scoring of histopathology endpoints suggested that MCLR toxicity drove NASH to a less fatty and more fibrotic state. In general, expression of genes involved in *de novo* lipogenesis and fatty acid esterification were altered in favor of decreased steatosis. The higher MCLR dose increased expression of genes involved in fibrosis and inflammation in the control and HFHC groups. These data suggest MCLR toxicity in the context of preexisting NASH may drive the liver to a more severe phenotype that resembles burnt-out NASH.

## 1. Introduction

Photoautotrophic cyanobacteria are increasingly prevalent and persistent in the environment as a result of anthropogenic activity and global warming [1,2]. Cyanobacteria produce a range of cyanotoxins as secondary metabolites that can cause neuro-, hepato-, renal-, or dermal-toxicity [3,4]. Exposure to and toxicity from these toxins has been reported in aquatic species, livestock, and human populations [3,5,6,7]. Microcystins are one family of secondary metabolites that bioaccumulate in aquatic animals and are transferred up the aquatic food web, ultimately reaching human consumption at different trophic levels [8,9]. Comprehensive reviews on cyanobacteria and their secondary metabolites have been published previously [5,6,10]. One of the most abundant, potent, and frequently studied hepatotoxic cyanotoxins is the cyclic heptapeptide microcystin-leucine-arginine (MCLR) [5]. Based on its toxicity and No Observable Adverse Effect Level (NOAEL), the World Health Organization (WHO) suggested a Tolerable Daily Intake (TDI) of 0.04 µg/kg body weight per day [11]. Recent estimates for MCLR exposure suggest people consuming fish and water from temperate lakes in North America and tropical lakes in Africa could exceed the TDI for MCLR consumption [8]. 

Multiple epidemiological and exposure studies have linked MCLR with liver damage. For example, an association between MCLR exposure and serum aspartate aminotransferase (AST) and alanine aminotransferase (ALT) was reported in a Chinese fisherman population exposed to TDI levels of MCLR (2.2–3.9 µg/day) [12]. In addition, a positive correlation between serum microcystin levels and hepatocellular carcinoma was reported in a case-control study in China [13], and several other studies reported an association between microcystin in drinking water and primary liver cancer [14,15,16]. Collectively, these data suggest a role of MCLR hepatotoxicity in liver disease, but the effects of MCLR toxicity in the context of preexisting liver disease are not known. 

The most common chronic liver disease in the United States is nonalcoholic fatty liver disease (NAFLD), and the worldwide prevalence of NAFLD is estimated to be ~25% [17]. NALFD is often referred to as the hepatic manifestation of metabolic syndrome because it is associated with obesity, insulin resistance, type 2 diabetes, and hypertriglyceridemia [17,18,19]. NAFLD progresses from steatosis to nonalcoholic steatohepatitis (NASH) and, eventually, to cirrhosis. Cryptogenic cirrhosis (i.e., cirrhosis from an unknown origin) is now commonly recognized as burnt-out NASH, or NASH that has advanced to severe fibrosis and inflammation coupled with decreased steatosis [17,19,20]. Given the high prevalence of NAFLD, there is great interest in identifying and charactering the environmental factors involved in progression of NAFLD to more severe stages. Recent evidence indicates that hepatotoxic environmental contaminants such as polychlorinated biphenyls, trichloroethylene, and chloroethanol may be involved in NAFLD pathogenesis [21,22,23,24,25,26,27]. 

A potential role of microcystins in NALFD development and progression has been previously suggested. Recently, a correlation between algal blooms and county level incidences of NAFLD in the United States was reported using a novel satellite imaging technique [28]. In addition, MCLR was reported to impact plasma insulin, glucose, triglycerides, and cholesterol levels, which are also associated with NAFLD [29]. Administration of the MCLR NOAEL (40 µg/kg) to mice via gavage every 48 h for 90 days induced a NASH-like phenotype characterized by liver steatosis and inflammation [30]. These data suggest that microcystin can cause toxicities resembling various aspects of NAFLD and may be involved in NAFLD pathogenesis. However, the effects of MCLR toxicity in the context of preexisting NAFLD are not known. The objective of this study was to determine how sub-chronic MCLR toxicity affects preexisting diet-induced NASH in rodents. Herein, we report that MCLR toxicity drives NASH to a less fatty and more fibrotic state, suggesting that MCLR-induced hepatotoxicity could increase the severity of NASH. 

## 2. Results and Discussion

The interaction between preexisting NASH with MCLR toxicity was determined using common dietary models of NASH and a common sub-chronic MCLR treatment design (dose level, route, duration, and frequency) [31,32,33,34,35,36,37,38,39]. MCLR (10 µg/kg or 30 µg/kg) or vehicle was administered via intraperitoneal injections every other day for 28 days to rats fed a control diet, a methionine and choline deficient (MCD) diet, or a high fat/high cholesterol (HFHC) diet to determine MCLR liver toxicity in healthy (control diet) versus NASH (MCD and HFHC diets) animals (Figure S1). The rationale for selecting the NASH diets is presented in Materials and Methods Section 4.1.1. 

### 2.1. Organ Weights, Body Weights, and Clinical Chemistry

Final body, liver, and spleen weights are shown in Table 1. Commonly reported changes in body weight from the MCD and the HFHC diets were observed in this study [40]. MCLR had no effect on body weight in any of the diet groups. Organ-to-body weight ratio is often used as an indicator of organ toxicity. Liver-to-body weight ratios decreased with 10 µg/kg and 30 µg/kg MCLR exposure in the control group and with 30 µg/kg MCLR exposure in the MCD group, primarily due to decreased liver weight. This MCLR effect on liver weight is opposite of hepatomegaly, which has been reported to occur in some cases of NASH, suggesting that the decreased liver-to-body weight ratio does not reflect clinical NASH [41]. Spleen-to-body weight ratios increased with 30 µg/kg MCLR exposure in the control and HFHC groups, primarily due to increased spleen weight. Published data for MCLR effects on spleen are limited, but our data are consistent with previous observations of spleen toxicity in fish and immunosuppression in isolated splenocytes [3,42,43]. This change in spleen weight may reflect increased portal hypertension and progression towards cirrhosis in these groups [44].

Plasma ALT is a potential biomarker for MCLR-induced liver toxicity and NALFD [12,33,45,46,47,48]. Plasma ALT was elevated in the MCD group after six weeks of diet prior to MCLR exposure (Figure S2). The 30 µg/kg MCLR exposed HFHC group was the only group to have elevated ALT after 14 days of MCLR exposure, suggesting this group may have developed MCLR-induced liver toxicity earlier than the other groups (Figure S2). At the end of the study (28 days of MCLR exposure), the 30 µg/kg MCLR dose increased ALT in control and HFHC groups (Figure S2). These data indicate ALT does not accurately reflect MCLR-induced liver toxicity in the MCD group, but could potentially be used to monitor MCLR-induced liver toxicity in the control and HFHC groups.

An analysis of plasma insulin and glucose levels revealed similarities between the control and HFHC groups in response to MCLR exposure (i.e., increased insulin and decreased glucose on day 29 after MCLR exposure) (Figure S3). It has been suggested that MCLR exposure can influence insulin and glucose levels and contribute to the occurrence of diabetes [29,49,50]. Evidence for hypoglycemia under severe or continuous MCLR toxicity has been reported. For example, severe hypoglycemia was common in the kidney dialysis patients from Brazil exposed to microcystins [51]. Also, mice receiving intraperitoneal injections of 20 µg/kg MCLR every other day for 16 weeks had decreased blood glucose levels [29]. Several studies also indicate that MCLR toxicity can impact insulin levels both in vitro and in vivo. For example, it has also been reported that treatment of INS-1 rat pancreatic β-cells increased insulin secretion at low MCLR dose (15n µM), but decreased insulin secretion at doses greater than 60 µM [52]. Also, Zhao et al. reported that blood insulin decreased after MCLR exposure [29]. The current data for increased insulin and decreased glucose after sub-chronic MCLR exposure, in combination with previously published data, indicate that MCLR toxicity consistently causes hypoglycemia and may have dose and time dependent effects on insulin. Plasma triglycerides levels decreased with MCLR exposure only in the control group on days 14 and 29, whereas plasma cholesterol levels increased with MCLR exposure only in the HFHC group on days 14 and 29 (Figures S3 and S4). The changes in cholesterol levels after MCLR exposure may play a role in NASH progression [53,54], but were not caused by changes in cholesterol synthesis genes (Figure S4). Collectively, these clinical biochemistry data further support that MCLR toxicity can alter metabolic homeostasis [12,50,51,52,55].

### 2.2. OATP1B2 and MCLR Western Blots

**Figure 1:**
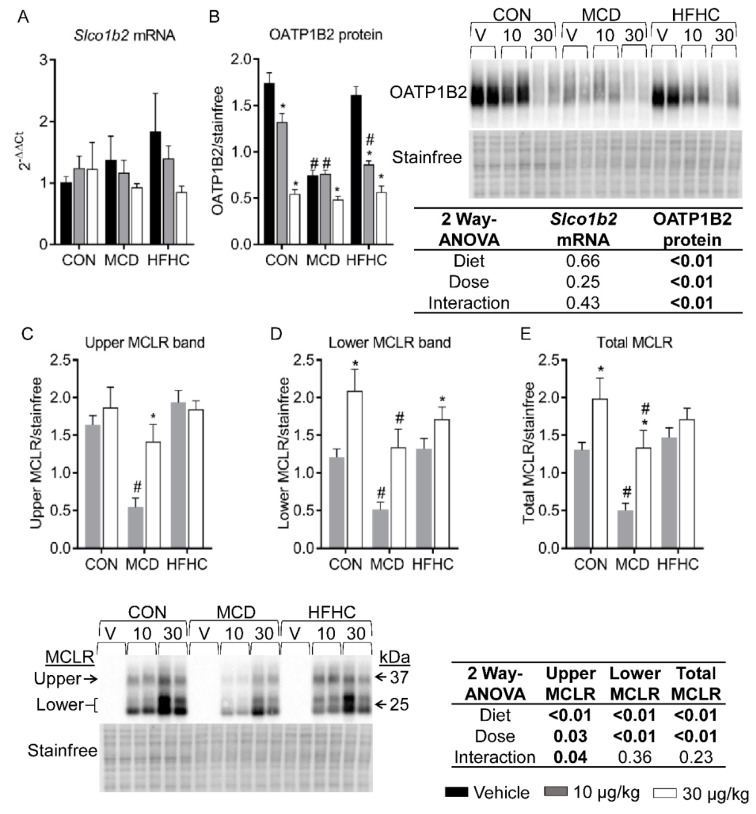
Liver *Slco1b2* mRNA (**A**) and OATP1B2 protein (**B**) and PP2A/C-bound MCLR (**C**–**E**). MCLR upper and lower bands were quantified by densitometry either individually (Panel (**C**,**D**)) or together (Panel (**E**)). Data represent mean ± SEM. N = 6 for each group. Two-way ANOVA *p*-values are shown in the table. * *p*-value < 0.05 versus respective vehicle according to Dunnett multiple comparison post-test (Panel (**A**,**B**)) and versus respective 10 µg/kg (Panel (**C**–**E**)) according to Sidak’s multiple comparison post-test. # *p*-value < 0.05 versus respective dose control according to Dunnett multiple comparison post-test (Panel (**A**–**E**)).

MCLR primarily causes hepatotoxicity because it is a preferential substrate for the hepatic OATP1B uptake transporters expressed on the hepatic sinusoidal membrane [56,57,58,59,60]. OATP1B2 is the rodent orthologue of human OATP1B1 and OATP1B3, and previous data show NASH alters OATP1B expression in human populations and rodent models [61,62,63,64]. No diet-nor MCLR-associated changes in *Slco1b2* mRNA expression were observed (Figure 1A). In contrast, OATP1B2 protein expression decreased with the MCD diet and both doses of MCLR decreased OATP1B2 protein expression in control and HFHC groups, although the 10 µg/kg MCLR dose decreased expression to a greater extent in the HFHC group compared to the control group (Figure 1B). For the MCD group, only the higher dose of MCLR decreased OATP1B2 protein expression (Figure 1B). These data suggest MCLR decreases OATP1B2 expression even at low doses and may act as a hepatoprotective mechanism to reduce MCLR uptake. 

MCLR is reported to induce some of its toxicity by covalently binding to the catalytic subunit of protein phosphatase 2A (PP2A) and inhibiting its activity [65,66]. In our study, MCLR affected PP2A catalytic subunit (PP2A/C) protein expression and PP2A activity only in the control diet group (Figure S5). The lack of major changes in PP2A activity may be related to the timing of collections (24 h after the last MCLR dose), but more research is needed to clarify this. Interestingly, a lower molecular weight PP2A/C band was observed only in the MCLR treated groups. A previous report investigating the degradation pathway for PP2A catalytic subunit involving the death inducing signaling complex, caspase-8, and Cul3, also observed upper and lower molecular weight PP2A bands in a western blot [67]. It is currently unclear whether this lower band represents a functional PP2A protein (Figure S5). 

PP2A/C-bound MCLR can be measured by western blot using an MCLR specific antibody [68]. As expected, MCLR bands were not detected in the vehicle treated animals from all diet groups, and MCLR bands were detected in the MCLR treated groups at the same molecular weights as the PP2A/C bands (Figure 1 C–E) [68,69]. Our data suggest the amount of PP2A/C-bound MCLR observed may be associated with the amount of MCLR that entered the hepatocytes through OATP1B2. The comparable expression levels of OATP1B2 between the MCD 10 µg/kg and MCD 30 µg/kg groups caused a ~2.7-fold increase in total PP2A/C-bound MCLR that corresponds with the 3-fold higher dose. Likewise, the higher expression levels of OATP1B2 in the control 10 µg/kg group compared to the control 30 µg/kg group potentially allowed the former group to take in more MCLR and caused only a 1.5-fold increase in total PP2A/C-bound MCLR from the lower to higher dose. The HFHC group diverges from this pattern; the 10 µg/kg HFHC group had similar levels of MCLR bound to PP2A/C as the 30 µg/kg group even though the OATP1B2 expression is comparable between the dose groups. These PP2A/C-bound MCLR data suggest OATP1B2 expression may be associated with how much MCLR is bound to PP2A/C within the hepatocyte.

### 2.3. Lipid Homeostasis

#### 2.3.1. Steatosis

**Figure 2:**
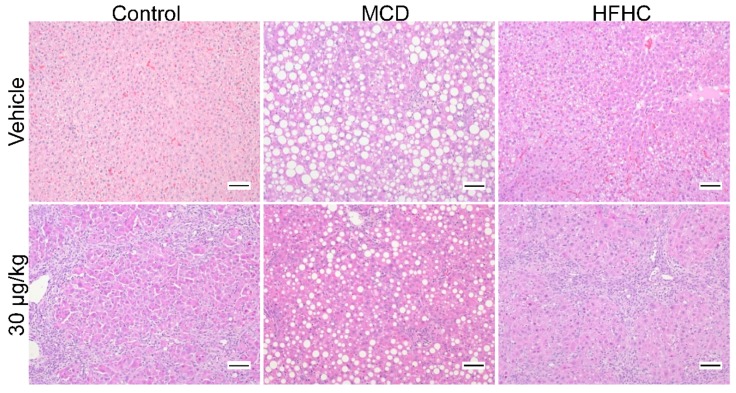
Representative H&E stained livers (magnification 100×). Intracellular lipid accumulation was observed as large to medium sized, round, clear vacuoles (macrovesicular vacuolation) in the MCD vehicle group, while small, round, clear vacuoles (microvesicular vacuolation) in a multifocal, zonal pattern were present in the HFHC vehicle group. Inflammatory cells (primarily lymphocytes) with deeply basophilic (blue) nuclei and scant to no eosinophilic (pink) cytoplasm were observed in every group, except for the control vehicle group. Scale Bars: 100 µm.

The progression of NASH to a more severe pathophysiological phenotype is characterized by the loss of steatosis and an increase in inflammation and fibrosis [20]. In our study, 30 µg/kg MCLR exposure decreased steatosis in both NASH models (Figure 2 and Table 2), suggesting that MCLR toxicity in the context of NASH may exacerbate the disease. We further characterized four integral pathways in hepatic lipid homeostasis: uptake of circulating lipids, *de novo* lipogenesis, fatty acid oxidation, and export of lipids [70].

#### 2.3.2. Fatty Acid Uptake and De Novo Lipogenesis

**Figure 3:**
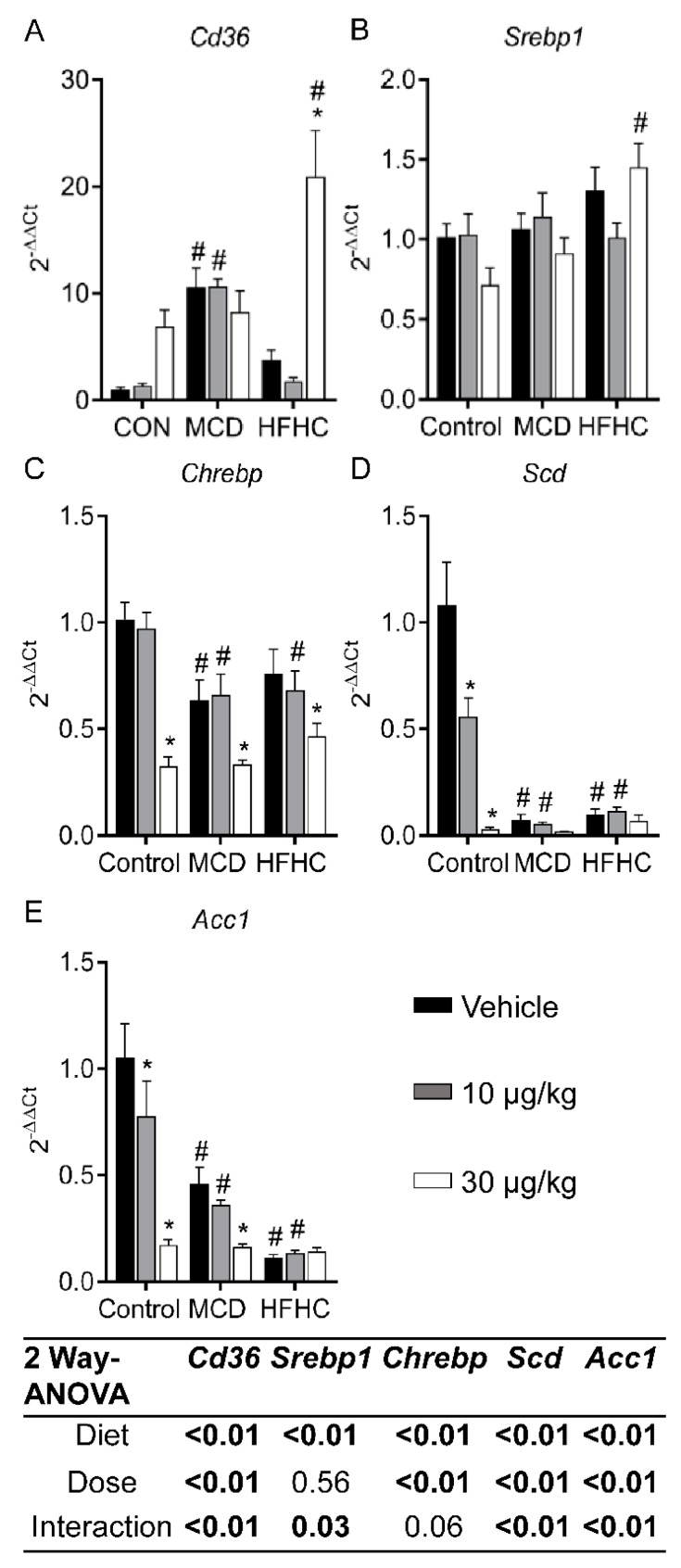
Liver *Cd36* (**A**), *Srebp1* (**B**), *Chrebp* (**C**), *Scd* (**D**), and *Acc1* (**E**) mRNA. Data represent mean ± SEM. N = 6 for each group. Two-way ANOVA *p*-values are shown in the tables. Dunnett multiple comparison post-test: * *p*-value < 0.05 versus respective vehicle; # *p*-value < 0.05 versus respective dose control.

Genes involved in fatty acid uptake and *de novo* lipogenesis are shown in Figure 3. Cluster of differentiation 36 (CD36) is a fatty acid translocase that plays an important role in facilitating the uptake of fatty acids [71,72]. It has been reported that deletion of *Cd36* in rodent models can exacerbate steatosis potentially due to increased *de novo* lipogenesis and decreased very low lipoprotein (VLDL) secretion [73]. In our study, MCLR exposure (30 µg/kg) increased *Cd36* expression in the HFHC group (Figure 3A), suggesting *Cd36* may be involved in decreasing steatosis after MCLR exposure in the HFHC model of NASH. 

Stearoyl-CoA desaturase (SCD) catalyzes the rate-limiting step in the conversion of saturated fatty acids to mono-unsaturated fatty acids. Total or liver specific knockout of *Scd* protects against obesity and steatosis, but increases liver injury and fibrosis [74,75]. *Scd* gene expression was lower in the MCD and HFHC groups compared to the control group, and 30 µg/kg MCLR decreased *Scd* in the control group (Figure 3D), suggesting that decreased expression of *Scd* in NASH and after MCLR toxicity may contribute to the progression of NASH to a less fatty and more fibrotic state. 

Acetyl CoA carboxylase-1 (ACC1) is another integral enzyme in *de novo* lipogenesis, and ACC1 inhibition is currently being explored as a target for NAFLD treatment by reducing steatosis [76]. Both MCLR doses decreased *Acc1* in the control group, and 30 µg/kg MCLR decreased *Acc1* gene expression in the MCD group (Figure 3E), suggesting that *Acc1* may play a role in the modulation of steatosis after MCLR exposure. 

Sterol regulatory element-binding protein 1 (SREBP1) and carbohydrate regulatory element-binding protein (ChREBP) are two key transcription factors involved in regulating the genes involved in *de novo* lipogenesis [70,77,78]. The HFHC diet increased expression of *Srebp1* and there was a significant difference between the control 30 µg/kg group and the HFHC 30 µg/kg group (Figure 3B). In contrast, 30 µg/kg MCLR exposure decreased *Chrebp* in all diet groups (Figure 3C). Liver-specific inhibition of *Chrebp* was reported to improve steatosis in ob/ob mice, suggesting a potential role of *Chrebp* in down-regulation of *de novo* lipogenesis genes and improvement in steatosis after MCLR exposure [79]. Collectively, these data suggest MCLR exposure may modulate steatosis through increased *Cd36* and decreased expression of *de novo* lipogenesis genes.

#### 2.3.3. Fatty Acid Oxidation Genes 

**Figure 4:**
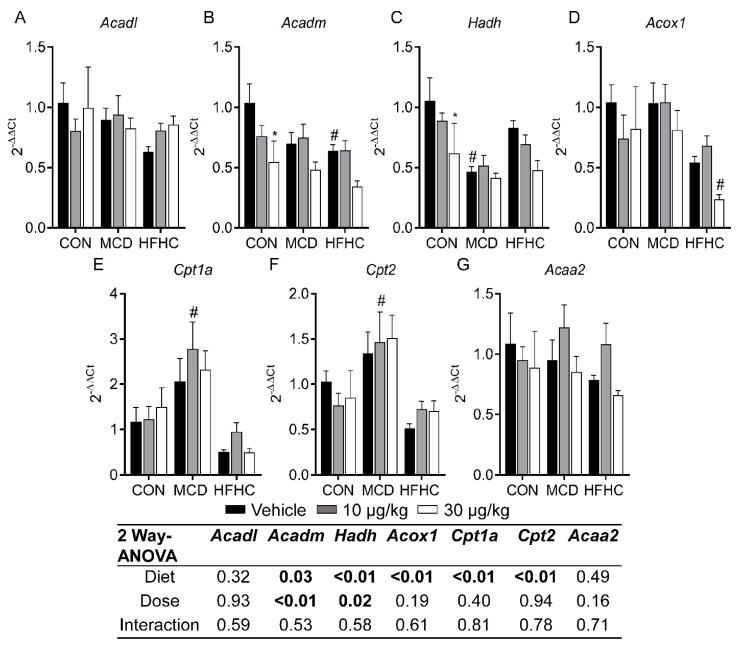
Liver *Acadl* (**A**), *Acadm* (**B**), *Hadh* (**C**), *Acox1* (**D**), *Cpt1a* (**E**), *Cpt2* (**F**), and *Acaa2* (**G**). Data represent mean ± SEM. N = 5 for each group. Two-way ANOVA *p*-values are shown in the tables. Dunnett multiple comparison post-test: * *p*-value < 0.05 versus respective vehicle; # *p*-value < 0.05 versus respective dose control.

Genes involved in fatty acid oxidation are shown in Figure 4. The NASH diets and MCLR toxicity had no effect on acyl-CoA dehydrogenase long chain (*Acadl*) and acetyl-CoA acyltransferase-2 (*Acaa2*) (Figure 4A,G). According to the two-way ANOVA p-values, the diet had a significant effect on acyl-CoA dehydrogenase medium chain (*Acadm*), hydroxyacyl-CoA dehydrogenase (*Hadh*), acyl-CoA oxidase-1 (*Acox1*), carnitine palmitoyltransferase-1A (*Cpt1*), and carnitine palmitoyltransferase-2 (*Cpt2*) (Figure 4B–G). MCLR exposure had a significant effect on only *Acadm* and *Hadh* gene expression, specifically decreasing expression of both in the control group (Figure 4B,C). These data suggest fatty acid oxidation may not play a major role in modulating steatosis after MCLR exposure. These data are congruent with previous results, indicating that the effects of NAFLD or MCLR toxicity on fatty acid oxidation are equivocal [70,80].

#### 2.3.4. Fatty Acid Esterification and Lipid Export

**Figure 5:**
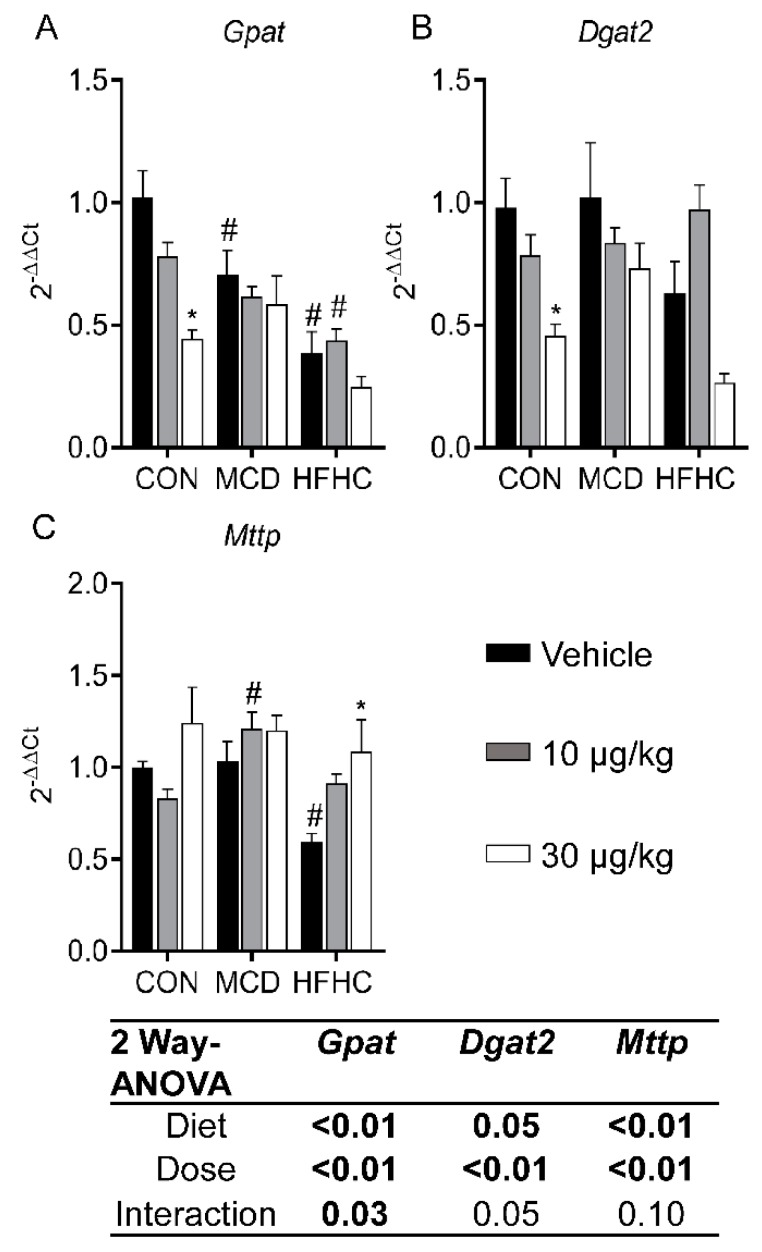
Liver *Gpat* (**A**), *Dgat2* (**B**), and *Mttp* (**C**). Data represent mean ± SEM. N = 5 for each group. Two-way ANOVA *p*-values are shown in the table. Dunnett multiple comparison post-test: * *p*-value < 0.05 versus respective vehicle; # *p*-value < 0.05 versus respective dose control.

Formation of triglycerides through hepatic fatty acid esterification is an important process in the development of steatosis and progression of NAFLD [81]. In this process, glycerol-3-phosphate acyltransferase (*Gpat*) is the rate-limiting enzyme, and diaclyglycerol O-acyltransferase 2 (*Dgat2*) catalyzes the final step [82,83]. In our study, MCLR significantly decreased *Gpat* and *Dgat2* expression (Figure 5A,B). These data are consistent with previous data indicating a deletion of either *Gpat* or *Dgat2* prevents steatosis in mouse models [81,84]. The microsomal triglyceride transfer protein (*Mttp*) gene is involved in producing lipoproteins responsible for lipid export from hepatocytes. *Mttp* expression increased after MCLR exposure (Figure 5C). A reduced function *Mttp* allele was recently found to be associated with pediatric NAFLD, potentially indicating increased expression of *Mttp* after MCLR exposure may contribute to the decrease in steatosis [85]. Collectively, these MCLR-induced changes in lipid homeostasis pathways are consistent with a reduction in hepatic fat accumulation and may be responsible for decreased steatosis in NASH. 

### 2.4. Inflammation 

**Figure 6:**
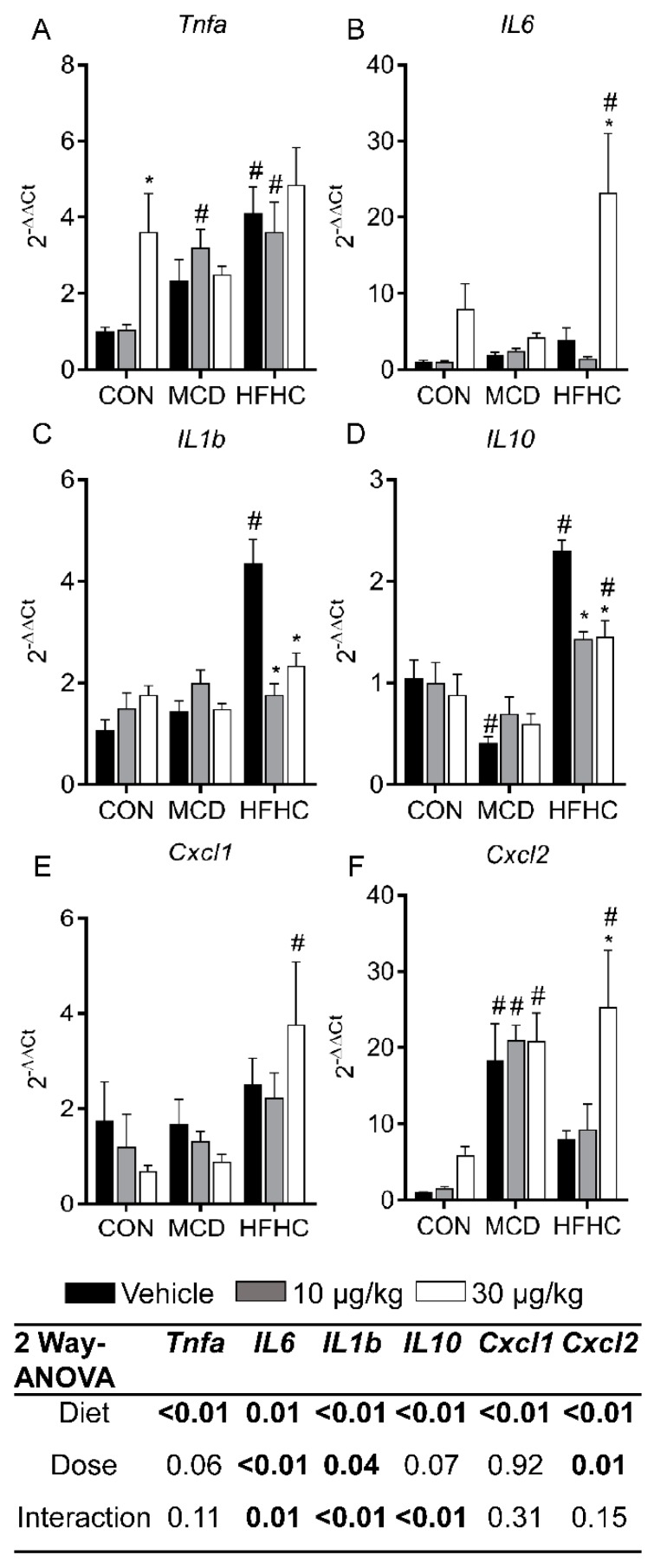
Liver *Tnf-α* (**A**), *IL6* (**B**), *IL1B* (**C**), *IL10* (**D**), *CxCl1* (**E**), and *CxCl2* (**F**). Data represent mean ± SEM. N = 6 for each group. Two-way ANOVA *p*-values are shown in the table. Dunnett multiple comparison post-test: * *p*-value < 0.05 versus respective vehicle; # *p*-value < 0.05 versus respective dose control.

NASH progression is hypothesized to advance through a multi-hit process, with lipid accumulation and inflammation acting as key pathogenic hits [86]. The two NASH models had significantly higher inflammation compared to the control group, and MCLR toxicity increased inflammation in all diet groups (Figure 2 and Table 2). These observations are consistent with known mechanisms of NASH and MCLR toxicity [30,46,63,87,88,89]. Although histopathological signs of inflammation after MCLR exposure were similar in all diet groups, a unique inflammatory response was observed in each diet group. First, the control group only showed increased tumor necrosis factor-α (*Tnf-α*) after MCLR exposure, whereas the HFHC group exhibited changes in interleukin-6 (*IL-6*) interleukin-1β (*IL-1*β), interleukin-10 (*IL-10*), and chemokine C-X-C motif ligand 2 (*CxCl2*) (Figure 6). In contrast, the MCD group did not have any MCLR-induced changes in cytokines or chemokines. All of the MCLR induced changes are consistent with a pro-inflammatory response [30,90] except for the decreased expression of *IL-1β* in the HFHC group. In fact, neutralization of this potent pro-inflammatory cytokine has been proposed as a target to inhibit the progression from simple fatty liver to NASH [86]. The reason for this discrepancy is unclear and requires further research to determine the mechanism behind it. In summary, MCLR toxicity caused hepatic inflammation in the control and the NASH groups, but our data suggest that MCLR toxicity can drive the progression of NASH to a more severe phenotype. 

### 2.5. Fibrosis

**Figure 7:**
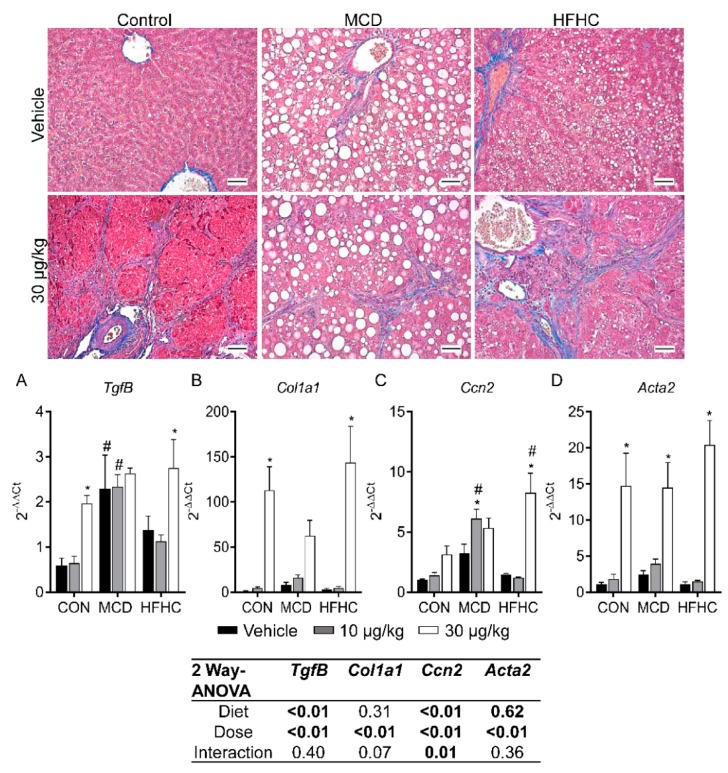
Representative Masson’s trichrome stained livers (magnification 100×). Intracellular lipid accumulation was observed as clear vacuoles within the cytoplasm (red). Collagen deposition (blue) was observed after MCLR treatment in all diet groups. Liver *TgfB* (**A**), *Col1a1* (**B**), *Ccn2* (**C**), and *Acta2* (**D**). Data represent mean ± SEM. N = 5 for each group. Two-way ANOVA *p*-values are shown in the tables. Dunnett multiple comparison post-test: * *p*-value < 0.05 versus respective vehicle; # *p*-value < 0.05 versus respective dose control. Scale bars: 100 µm.

Fibrosis is another key feature of NAFLD progression and is associated with more severe liver damage and advanced disease. MCLR is also known to induce liver fibrosis in pre-clinical models and has been observed in people after acute exposure to microcystins [39,51,91]. In the current study, MCLR toxicity induced fibrosis in all three diet groups, although there were some notable differences between the groups (Figure 2 and Figure 7, Table 2). For example, the MCD group was susceptible to MCLR-induced fibrosis at the low-dose of MCLR unlike the HFHC and control groups, which were refractory at this dose (Table 2). In addition, the control group exhibited the highest severity of MCLR-induced fibrosis compared to the NASH models. These data suggest that MCLR-induced fibrosis may be more severe in a healthy liver in comparison to a NASH liver. An examination of genes involved in fibrosis revealed the 30 µg/kg MCLR dose increased expression of transforming growth factor-β (*Tgfβ*), collagen type 1 alpha 1 chain (*Col1a1*), cellular communication network factor 2 (*Ccn2*), and smooth muscle actin alpha 2 (*Acta2*) gene expression in the control and HFHC groups (Figure 7). *Tgfβ* and *Ccn2* expression was already elevated in the MCD group, and the only MCLR-induced change in the MCD groups was an increase in *Ccn2* at the 10 µg/kg dose. Collectively, these data suggest a blunted MCLR-induced fibrotic response in the NASH groups compared to the control group, although the susceptibility threshold may be lower in the MCD group compared to control and the HFHC groups. In NAFLD patients, fibrosis is a predictor of clinical outcome, suggesting that MCLR-induced fibrosis may affect the long-term health of NAFLD patients [92]. Our data are also consistent with previous data indicating that changes in fibrosis do not correlate with ALT levels, indicating that ALT may not be a reliable marker of MCLR-induced fibrosis in the context of NASH [92]. Similar to the inflammation results, these data also suggest MCLR toxicity may drive the progression of NASH to a more severe phenotype. 

## 3. Conclusions

MCLR is a ubiquitous cyanotoxin that has been studied extensively for its hepatotoxic effects in rodent models with healthy livers (i.e., no preexisting liver disease) [11,30,50,93,94]. It was previously reported that MCLR toxicity can produce a NASH-like phenotype in the absence of a NASH-inducing diet [30]. Our novel study demonstrates that sub-chronic MCLR exposure in the context of preexisting diet-induced NASH can produce a more severe NASH phenotype as indicated by decreased steatosis, increased inflammation, and increased fibrosis. These pathological features are associated with development of what is referred to as burnt-out NASH [95]. Burnt-out NASH is now recognized as a component of cryptogenic cirrhosis, which is a growing cause for orthotopic liver transplantation [96]. Toxin exposure is one of the etiological factors for consideration when diagnosing cryptogenic cirrhosis, and our study demonstrates that MCLR exposure may contribute to the clinical burden of cryptogenic cirrhosis [96]. This research also demonstrates subtle differences in susceptibility to MCLR hepatotoxicity, but overall suggests that healthy and NASH livers have similar susceptibility. Finally, this research is timely because the prevalence of both algal blooms and NASH are growing, thus increasing the likelihood of MCLR exposures in populations with preexisting liver disease. 

## 4. Materials and Methods

### 4.1. Sub-Chronic Toxicity Study

#### 4.1.1. Selection of Diet-Induced NASH Models 

Three diets were utilized in this study: a control diet (Dyets Inc., Bethlehem, PA, USA, Cat. 518754), an MCD diet (Dyets Inc. Cat. 518810), and a HFHC diet (Research Diets, New Brunswick, NJ, USA, Cat. D06061401). These diets were selected based on their strengths and weaknesses in recapitulating features of clinical NASH. The control diet produces a healthy phenotype, while the MCD and HFHC diets are established dietary models used to produce a NASH phenotype. The two NASH diets have their respective strengths and weaknesses. The MCD diet replicates NASH liver pathology and is reported as a good model to study NAFLD progression because it causes steatosis, inflammation, oxidative stress, and fibrosis in a short period of time (less than 6 weeks) [46,97,98,99]. The HFHC diet also produces a robust replication of NASH liver pathology, and, in contrast to the MCD diet, also represents a more clinically relevant model of NASH development (i.e., over-nutrition) [31,32,33]. A previous report indicates that Sprague Dawley rats fed the MCD or HFHC diet for 8 weeks have NASH activity scores ≥ 4, indicating a positive NASH diagnosis [40]. Another strength of both NASH diets is that they replicate the changes in xenobiotic transporters observed in clinical NASH, specifically the organic anion transporting polypeptide-1B (OATP1B) transporters that are important for MCLR uptake [40].

#### 4.1.2. MCLR Exposure Protocol

The MCLR exposure scenario (i.e., dose, route, duration, and frequency) is commonly utilized to study sub-chronic MCLR toxicity and was selected to isolate hepatic exposure and toxicity mechanisms from those occurring in the gastrointestinal tract [36,37,38,39,40,41]. Eight-week-old male Sprague-Dawley rats were purchased from Envigo (Huntingdon, Cambridgeshire, UK). MCLR was purchased from Cayman Chemicals (Ann Arbor, MI, USA). Handling, care, and maintenance of the animals was done in the Association for the Assessment of Laboratory Animal Care International accredited Program of the Laboratory Animal Resources facility of Washington State University, Spokane. All animals were maintained in 12-h light and dark cycles for the duration of the study. The experimental protocol was approved by the Institutional Animal Care and Use Committee at Washington State University, approval code is 04937 and the approval date was 14 December 2016. 

Animals were divided into three groups (n = 18 per group) and fed one of the diets listed above for 6 weeks, at which point each diet group was further divided into three treatment groups (n = 6 per group): vehicle (saline with 0.09% ethanol), 10 µg/kg MCLR, or 30 µg/kg MCLR (Figure S1). The animals continued consuming their respective diets and began receiving intraperitoneal injections of vehicle or MCLR every 48 h for an additional 4 weeks (10 weeks total of dietary treatment). Blood was collected from the tail vein into heparinized tubes prior to the first MCLR dose (day 0), 24 h after the seventh dose (day 14), and 24 h after the last dose (day 29). Blood was centrifuged at 10,000× *g* for 5 min at 4 °C and plasma was removed and stored at −80 °C until further analysis. Animals were housed in metabolic cages starting 24 h prior to, and 24 h after, the first and last MCLR doses (48-h housing duration each time). Twenty-four hours after the final MCLR injection, animals were euthanized by carbon-dioxide asphyxiation and tissues were collected. Portions of tissues were formalin fixed for histopathological scoring or snap frozen for total RNA isolation or protein isolation.

### 4.2. mRNA Expression 

Total RNA was extracted from rat liver using TRIzol reagent (Thermo Fischer Scientific, Waltham, MA, USA) according to the manufacturer’s protocol. RNA concentrations were determined using a nano-drop, and RNA integrity was confirmed by agarose gel electrophoresis. iScript cDNA synthesis kit (Bio-Rad) was used for cDNA synthesis from total RNA, and SYBR green master mix (Bio-Rad, Hercules, CA, USA) was used for real time quantitative PCR analysis as per the manufacturer’s protocol. Primers (Supplementary Table S1) were purchased from Sigma (St. Louis, MO, USA) for the following genes: Carbohydrate-responsive element-binding protein (*Chrebp*), Sterol regulatory element-binding protein 1 (*Srebp1*), Acetyl CoA Carboxylase 1 (*Acc1*), stearoyl CoA desaturase (*Scd*), Cluster of differentiation 36 (*Cd36*), Transforming growth factor β (*Tgfβ*), Collagen type 1 alpha 1 (*Col1a1*), Cellular communication network-2 (*Ccn2*), Actin, alpha 2, smooth muscle, aorta (*Acta2*), C-X-C motif chemokine ligand 2 (*Cxcl2*), Tumor necrosis factor-α (*Tnf-α*), Interleukin -6 (*IL6*), Interleukin 1-Beta (*IL1B*), Interleukin 10 (*IL10*), C-X-C motif chemokine ligand 1 (*Cxcl1*), acyl-CoA dehydrogenase long chain (*Acadl*), acyl-CoA oxidase-1 (*Acox1*), carnitine palmitoyltransferase-1A (*Cpt1*) and carnitine palmitoyltransferase-2 (*Cpt2*) and acetyl-CoA acyltransferase-2 (*Acaa2*), Acyl-CoA dehydrogenase medium chain (*Acadm*) and hydroxyacyl-CoA dehydrogenase (*Hadh*), Glycerol-3-phosphate acyltransferase (*Gpat*), Diaclyglycerol O-acyltransferase 2 (*Dgat2*), Microsomal triglyceride transfer protein (*Mttp*), Ubiquitin C (*Ubc*), Glyceraldehyde-3-phosphate dehydrogenase (*Gapdh*), and Beta-2 microglobulin (*β2M*). Primers for *Slco1b2* was purchased from Integrated DNA Technologies (IDT) (Coralville, Iowa, USA). The expression for the genes of interest were normalized to the average expression of three housekeeping genes (*Ubc, Gapdh* and *β2M*). 

### 4.3. Histopathological Analysis

Formalin fixed liver tissues were paraffin embedded, stained with hematoxylin and eosin (H&E), and analyzed by a board-certified veterinary pathologist. Stained sections were incidence and severity scored using an established rodent NASH system [100] with endpoints including lipid accumulation, necrosis, inflammation, biliary hyperplasia, and fibrosis. Pathology scores were as follows: 0, no significant lesions (0%); 1, minimal (<10%); 2, mild (10%–25%); 3, moderate (25%–40%); 4, marked (40%–50%); 5, severe (>50%). A second set of formalin fixed tissues were stained with Masson trichrome stain and were used for qualitative analysis of fibrosis based on overall amount and intensity of positive staining.

### 4.4. Protein Preparations

Approximately 50 mg of liver tissue was homogenized with a handheld homogenizer in 1 mL of NP40 lysis buffer with protease inhibitors. The homogenized samples were agitated at 4 °C for 2 h followed by centrifugation at 10,000× *g* at 4 °C for 1 h. Supernatants were collected, avoiding the lipid layer. Protein concentrations were determined using the Pierce BCA Protein Quantification Assay kit (Thermo Fisher Scientific, Waltham, MA, USA) according to the manufacturer’s protocol. 

### 4.5. Immunoblot Protein Analysis

Tissue lysates (20 µg/well) were prepared in Laemmli sample buffer with 2.5% BME and heated at 37 °C for 30 min. Protein was transferred from the gel to polyvinylidene fluoride (PVDF) membrane using the Trans-Blot Turbo Transfer System at 25 V and 1.0 A for 30 min. Following transfer, the membranes were imaged under UV to capture Stain-Free image used for protein normalization. The blots were then blocked with 5% non-fat dry milk in Tris-base buffered saline-Tween 20 (TBS-T) for 1 h at room temperature, then incubated with primary antibody overnight at 4 °C. Membranes were probed for OATP1B2 (1:1000 dilution; Santa Cruz Biotechnology, Santa Cruz, CA, USA, Cat. 376904) and MCLR (1:2000 dilution; Enzo, Farmingdale, NY, USA, Cat. 89154-022). The blots were incubated with secondary antibody in 5% non-fat dry milk in TBS-T for 1 h at room temperature. Densitometry was performed using Image Lab (Bio-Rad, Standard Edition, Version 6.0.0 build 25). Proteins of interest were normalized to total protein as captured by Stain-Free image. Total protein normalization is a commonly accepted technique for protein densitometry analysis instead of the single-protein loading control [101].

### 4.6. Statistical Analysis

All results are represented as mean ± SEM. All data, except the histopathological data, were analyzed by two-way ANOVA statistical analysis with diet and dose constituting the main two factors. A Dunnett multiple comparison post-test was performed to determine statistical differences between different treatment groups. Histopathological data were rank ordered before statistical analysis. All analyses were carried out using GraphPad Prism software. (GraphPad Software, Inc., La Jolla, CA, USA).

## Figures and Tables

**Table 1:** Final body and tissue weights with accompanying tissue-to-body weight ratios.

Group	Body (g)	Liver (g)	Spleen (g)	Liver: Body	Spleen: Body
Control					
Vehicle	379 ± 7.7	15.38 ± 0.4	0.75 ± 0.03	0.041 ± 0.001	0.002 ± 0.0001
10 µg/kg	357 ± 9	**12.08 ± 0.62** *	0.69 ± 0.02	**0.034 ± 0.002** *	0.002
30 µg/kg	375 ± 11.8	**12.07 ± 0.52** *	**1.11 ± 0.07** *	**0.032 ± 0.002** *	**0.003 ± 0.0001** *
MCD					
Vehicle	**179 ± 5.6** ^#^	**8.39 ± 0.33** ^#^	**0.36 ± 0.01** ^#^	**0.047 ± 0.001** ^#^	0.002
10 µg/kg	**187 ± 4.4** ^#^	**8.40 ± 0.19** ^#^	**0.40 ± 0.02** ^#^	**0.045 ± 0.001** ^#^	0.002 ± 0.0001
30 µg/kg	**177 ± 3.6** ^#^	**7.21 ± 0.2** ^#^	**0.42 ± 0.03** ^#^	**0.041 ± 0.001** *^#^	0.002 ± 0.0001
HFHC					
Vehicle	**408 ± 6.8** ^#^	**18.49 ± 1.1** ^#^	**1.13 ± 0.1** ^#^	0.045 ± 0.003	**0.003 ± 0.0002** ^#^
10 µg/kg	**420 ± 6.8** ^#^	**18.07 ± 0.39** ^#^	**0.89 ± 0.05** *	**0.043 ± 0.001** ^#^	**0.002 ± 0.0001** *
30 µg/kg	**406 ± 10.3** ^#^	**20.20 ± 1.51** ^#^	**2.15 ± 0.16** *^#^	**0.050 ± 0.003** ^#^	**0.005 ± 0.0004** *^#^
Two-way ANOVA					
Diet	<0.01 (97%)	<0.01 (84%)	<0.01 (55%)	<0.01 (44%)	<0.01 (26%)
Dose	0.91 (<1%)	0.10 (1%)	<0.01 (21%)	0.04 (6%)	<0.01 (36%)
Interaction	0.15 (<1%)	<0.01 (4%)	<0.01 (16%)	<0.01 (14%)	<0.01 (24%)

Data represent mean ± SEM. N = 6 for each group. Bold values with (*) indicate *p* value < 0.05 versus respective vehicle group within each diet group according to a Two-way ANOVA Dunnett multiple comparison post-test. Bold values with (#) indicate *p* value < 0.05 versus respective control group within each dose group according to a Two-way ANOVA Dunnett multiple comparison post-test. The percentage of the total variance associated with each factor (diet, dose, interaction) are shown in parentheses next to the Two-way ANOVA *p*-values.

**Table 2:** Liver histopathology scores.

Group	Steatosis	Inflammation	Fibrosis	Biliary Hyperplasia
Control				
Vehicle	0	0	0	0
10 µg/kg	0	0.2 ± 0.2	0	0
30 µg/kg	0	**2.2 ± 0.2** *	**3.2 ± 0.3** *	**1.7 ± 0.2** *
MCD				
Vehicle	**3.5 ± 0.3** ^#^	**1** ^#^	0	0.2 ± 0.2
10 µg/kg	**3.3 ± 0.2** ^#^	**2** *^#^	**1** *^#^	**1** *^#^
30 µg/kg	**2.2 ± 0.2** *^#^	**1.8 ± 0.2** *	**1.7 ± 0.2** *^#^	**1.8 ± 0.2** *
HFHC				
Vehicle	**2.6 ± 0.2** ^#^	**1.3 ± 0.2** ^#^	0.2 ± 0.2	0.3 ± 0.2
10 µg/kg	**2.3 ± 0.3** ^#^	**0.5 ± 0.3** *	0	0.2 ± 0.2
30 µg/kg	**1.8 ± 0.2** *^#^	**2.2 ± 0.2** *	**2.3 ± 0.2** *^#^	**2.0 ± 0.2** *
Two-way ANOVA				
Diet	<0.01 (82%)	<0.01 (15%)	0.19 (1%)	<0.01 (4%)
Dose	<0.01 (5%)	<0.01 (41%)	<0.01 (77%)	<0.01 (72%)
Interaction	0.02 (3%)	<0.01 (26%)	<0.01 (14%)	0.01 (6%)

Data represent mean ± SEM. N = 6 for each group. Two-way ANOVA Dunnett multiple comparison post-test: bold values with (*) indicate *p*-value < 0.05 versus respective vehicle group within each diet group; bold values with (#) indicate *p* value < 0.05 versus respective control group within each dose group. The percentage of the total variance associated with each factor (diet, dose, interaction) are shown in parentheses next to the Two-way ANOVA *p*-values.

## Supplementary Materials

The following are available online at https://www.mdpi.com/2072-6651/11/7/398/s1, Materials and methods: Plasma chemistry, mRNA expression, PP2A immunoblotting and activity assay. Figure S1: Sub-chronic MCLR exposure study design. Figure S2: Plasma ALT. Figure S3: Plasma insulin (**A**), glucose (**B**), and triglycerides (**C**). Figure S4: Plasma cholesterol (**A**) and liver *Srebp2* (**B**), *Sqe* (**C**), and *Hmgcr* (**D**) mRNA. Figure S5: Liver PP2A protein expression (**A**–**C**) and PP2A activity. Table S1: list of primers.
