# Supplementary material for: Sub-Chronic Microcystin-LR Liver Toxicity in Preexisting Diet-Induced Nonalcoholic Steatohepatitis in Rats
Source: Toxins (Basel). 2019 Jul 9;11(7):398. doi: 10.3390/toxins11070398 (PMC6669744; doi:10.3390/toxins11070398)
Supplement: Supplementary file 1 [file toxins-11-00398-s001.pdf]

Supplementary Materials: Sub-Chronic Microcystin-LR Liver Toxicity in Preexisting Diet-Induced Nonalcoholic Steatohepatitis in Rats

Tarana Arman, Katherine D. Lynch, Michelle L Montonye, Michael Goedken and John D. Clarke

S1. Supplementary Materials and Methods:

S1.1. Plasma Chemistries

Plasma chemistries were analyzed using commercially available kits according to the manufacturer's protocols. Triglyceride (Cat. 10010303), glucose (Cat. 10009582), and alanine transaminase (ALT) (Cat. 700260) quantification was performed with colorimetric assays and cholesterol (Cat. 10007640) quantification was performed with a fluorometric assay (Cayman Chemicals). Insulin quantification was performed with an ELISA kit (Millipore, Cat. EZRMI-13K).

S1.2. mRNA Expression

Total RNA was extracted from rat liver using TRIzol reagent (Thermo Fischer Scientific) according to the manufacturer's protocol. RNA concentrations were determined using a nano-drop and RNA integrity was confirmed by agarose gel electrophoresis. iScript cDNA synthesis kit (Bio-Rad) was used for cDNA synthesis from total RNA and SYBR green master mix (Bio-Rad) was used for real time quantitative PCR analysis as per the manufacturer's protocol. Primers (Supplementary Table 1) were purchased from Sigma (St. Louis, Missouri, USA) for the following genes: Sterol regulatory element-binding protein 2 *(Srebp2),* 3-hydroxy-3-methylglutaryl-coenzyme A reductase *(Hmgcr),* Squalene exposidase *(Sqe).* The expression for the genes of interest were normalized to the average expression of three housekeeping genes (*Ubc, Gapdh* and *β2M*).

S1.3. PP2A/C Immunoblotting

Tissue lysates (20 µg/well) were prepared in Laemmli sample buffer with 2.5% BME and heated at 37°C for 30 minutes. Protein was transferred from the gel to polyvinylidene fluoride (PVDF) membrane using the Trans-Blot Turbo Transfer System at 25 V and 1.0 A for 30 minutes. Following transfer, the membranes were imaged under UV to capture Stain-Free image used for protein normalization. The blots were then blocked with 5% non-fat dry milk in Tris-base buffered saline-Tween 20 (TBS-T) for 1 hour at room temperature and incubated with primary antibody overnight at 4°C. Membranes were blotted for PP2A subunit C (1:2,000 dilution; Millipore, Burlington, Massachusetts, USA, Cat. 05-421). The blots were incubated with secondary antibody in 5% non-fat dry milk in TBS-T for 1 hour at room temperature. Densitometry was performed using Image Lab (Bio-Rad, Standard Edition, Version 6.0.0 build 25). Proteins of interest were normalized to total protein as captured by Stain-Free image.

S1.4. PP2A Activity Assay

Liver tissues for the PP2A activity assay were prepared by grinding the frozen tissues using a mortar and pestle. 100 mg of ground tissue was weighed out and 1ml of NP40 lysis buffer was added to it. The tubes were placed in cold tissue-lyser blocks along with 3 metal beads in each tube. The cells were lysed twice at 30 hertz for 3 minutes with a rest period in ice for 5 minutes in between. The supernatant was centrifuged at 15000g for 10 minutes at 4°C. Supernatants were collected from the centrifuged samples and care was taken to avoid the lipid layer. Protein concentrations were determined using the Pierce BCA Protein Quantification Assay kit (Thermo Fisher Scientific, Waltham, Massachusetts, USA) according to the manufacturer's protocol. PP2A activity was

measured in these samples using a commercially available fluorometric assay kit (ThermoFisher Scientific, Waltham, MA, USA, Cat R33700) according to manufacturer's protocol.

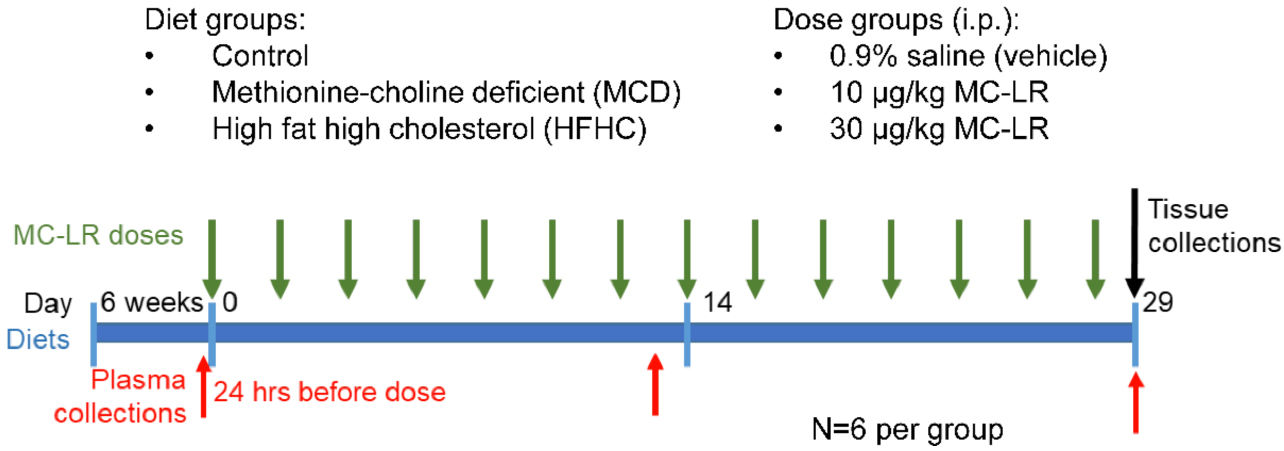

Figure S1. Sub-chronic MCLR exposure study design.

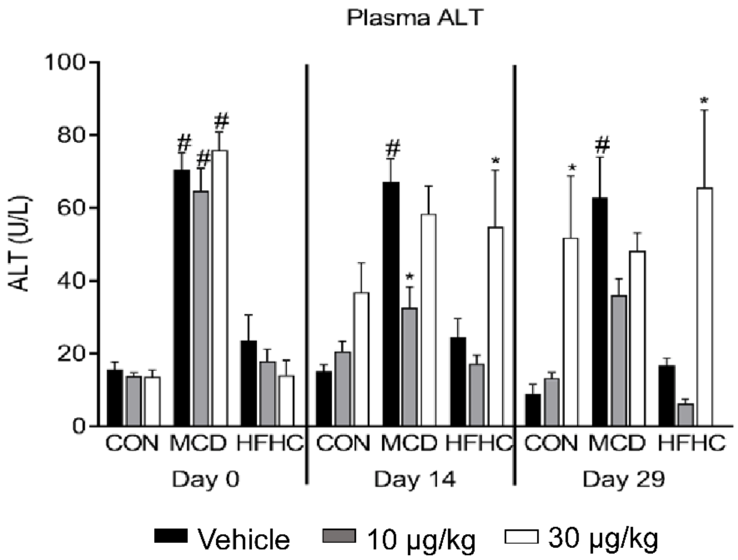

2 Way-ANOVA	Day 0	Day 14	Day 29
Diet	**<0.01**	**<0.01**	**<0.01**
Dose	0.46	**<0.01**	**<0.01**
Interaction	0.38	**0.03**	**0.01**

Figure S2. Plasma ALT. Data represent mean ± SEM. N = 6 for each group. Two-way ANOVA *p*-values are shown in the tables. Dunnett multiple comparison post-test: **p*-value <0.05 versus respective vehicle; #*p*-value <0.05 versus respective dose control.

A

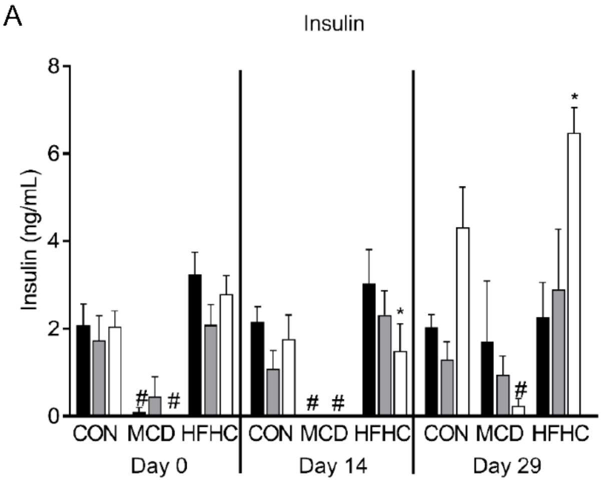

2 Way-ANOVA	Day 0	Day 14	Day 29
Diet	**<0.01**	**<0.01**	**<0.01**
Dose	0.53	0.17	**0.01**
Interaction	0.46	0.33	**0.01**

B

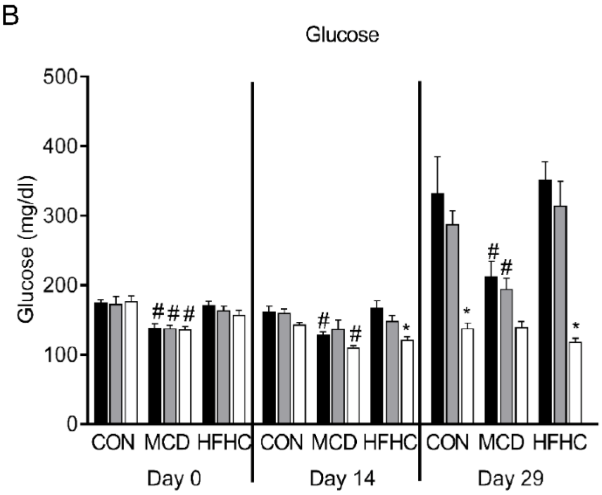

2 Way-ANOVA	Day 0	Day 14	Day 29
Diet	**<0.01**	**<0.01**	**<0.01**
Dose	0.62	**<0.01**	**<0.01**
Interaction	0.75	0.21	**0.02**

C

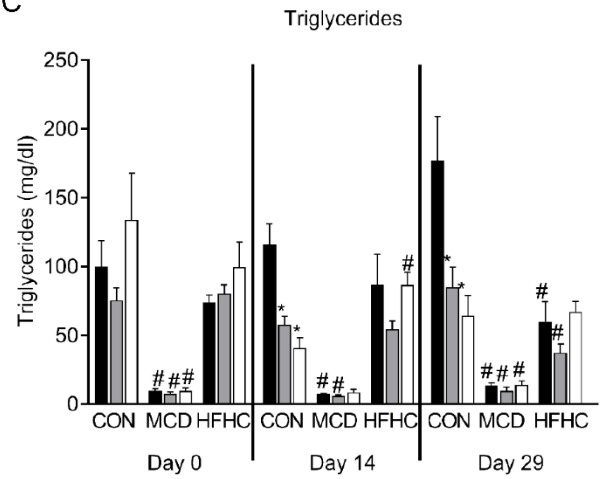

2 Way-ANOVA	Day 0	Day 14	Day 29
Diet	**<0.01**	**<0.01**	**<0.01**
Dose	0.08	**<0.01**	**<0.01**
Interaction	0.40	**<0.01**	**<0.01**

Vehicle 10 µg/kg 30 µg/kg

Figure S3. Plasma insulin (**A**), glucose (**B**), and triglycerides (**C**). Data represent mean ± SEM. N = 6 for each group. Two-way ANOVA *p*-values are shown in the tables. Dunnett multiple comparison post-test: **p*-value <0.05 versus respective vehicle; #*p*-value <0.05 versus respective dose control.

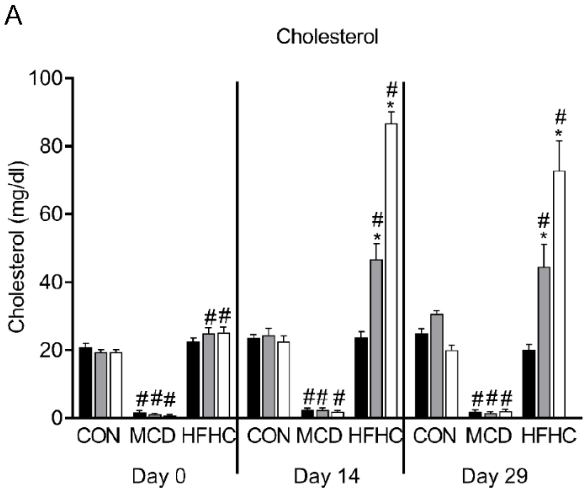

**2 Way-ANOVA**	**Day 0**	**Day 14**	**Day 29**
Diet	**<0.01**	**<0.01**	**<0.01**
Dose	0.99	**<0.01**	**<0.01**
Interaction	0.21	**<0.01**	**<0.01**

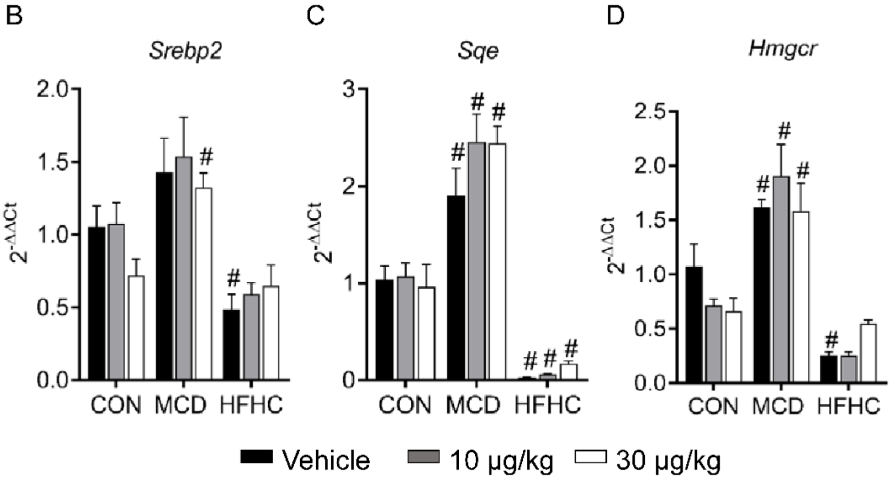

**2 Way-ANOVA**	***Srebp2***	***Sqe***	***Hmgcr***
Diet	**<0.01**	**<0.01**	**<0.01**
Dose	0.46	0.34	0.93
Interaction	0.48	0.45	0.08

Figure S4. Plasma cholesterol (**A**) and liver *Srebp2* (**B**), *Sqe* (**C**), and *Hmgcr* (**D**) mRNA expression. Data represent mean ± SEM. N = 5 for each group. Two-way ANOVA *p*-values are shown in the tables. Dunnett multiple comparison post-test: **p*-value <0.05 versus respective vehicle; #*p*-value <0.05 versus respective dose control.

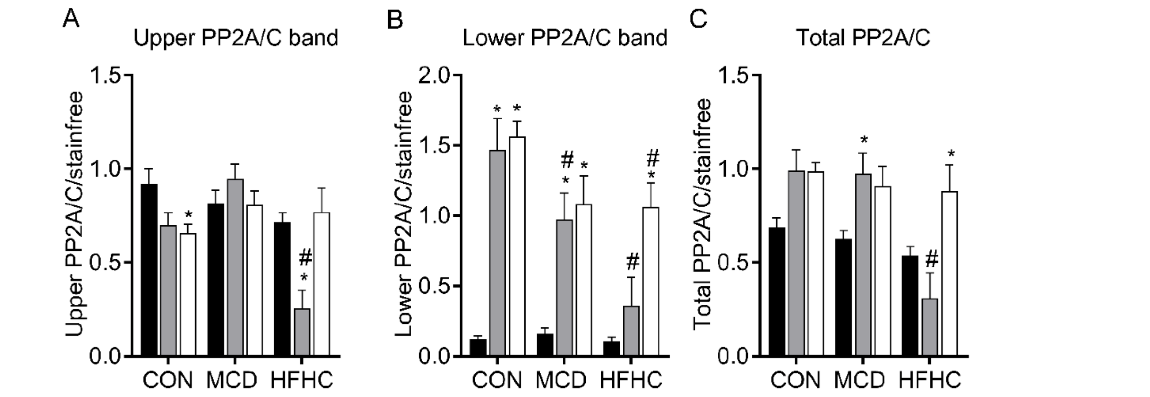

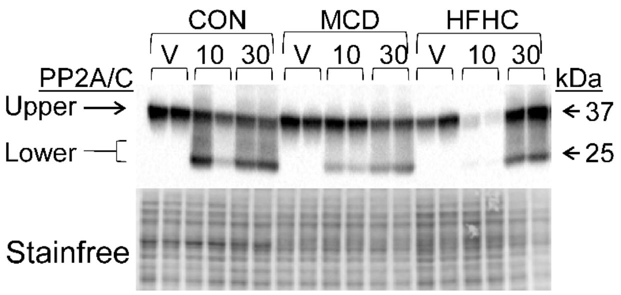

2 Way-ANOVA	Upper PP2A/C	Lower PP2A/C	Total PP2A/C
Diet	**<0.01**	**<0.01**	**<0.01**
Dose	**0.03**	**<0.01**	**<0.01**
Interaction	**<0.01**	**0.01**	**<0.01**

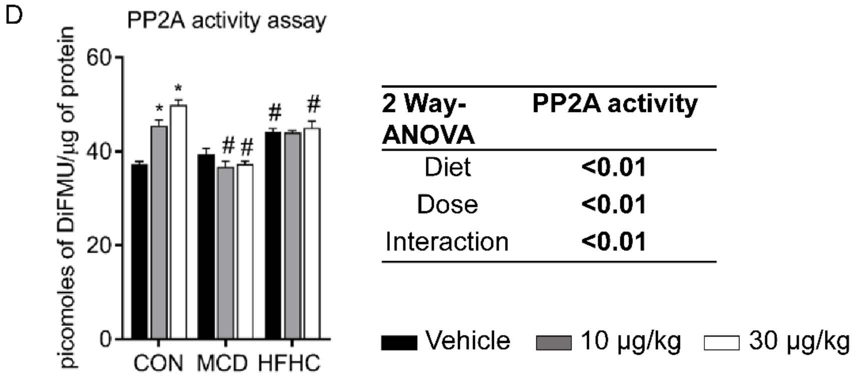

2 Way-ANOVA	PP2A activity
Diet	**<0.01**
Dose	**<0.01**
Interaction	**<0.01**

Figure S5. Liver PP2A/C protein expression (**A**–**C**) and PP2A activity (**D**) PP2A/C blot had upper and lower bands that were quantified by densitometry either individually (Panel **A** and **B**) or together (Panel **C**). Data represent mean ± SEM. N = 6 for each group. Two-way ANOVA *p*-values are shown in the tables. Dunnett multiple comparison post-test: **p*-value <0.05 versus respective vehicle; #*p*-value <0.05 versus respective dose control.

Table 1. List of primers.

Gene	NCBI ID	Forward sequence (5'–3')	Reverse sequence (5'–3')
Slco1b2	NM_031650.3	GGGTGAATGCCCAAGATACA	GCTGGTGACAGACCACTTAAT A
Cd36	NM_031561.2	TGGACTTGTACTCTCTCCTCG	GCCAGGACAGCACCAATAAC
Srebp1	NM_001276707.1	ACGTGGGTCTCCTCCGAAGC	AGCATGTCTTCGATGTCGGTCAAGAG
Chrebp	NM_133552.1	TCAGAAGCAGATGAGCACCG	CTTCCCTGCTGGACTTACGG
Scd	NM_139192.2	ACAACTACCATCACGCCTTCC	TGGAACAGGAACTCAGAAGCCC
Acc1	NM_022193.1	ATTGGGGCTTACCTTGTCCG	TGCATTATCTGGATGCCCCC
Acadl	NM_012819.1	GCACAAAAGAACAGATCGAG	GAGAATCCAATCACTCCCAG
Acadm	NM_016986.2	CCAGAGAGGAAATAATCCCG	AAACACGCATCAAAAGTTCC
Hadh	NM_057186.2	CTCTGACGTAATCTCTTGGC	GTGAATGCGGCTAATGATTG
Acox1	NM_017340.2	AGAAGATGAGGGAATATGGC	GGAGTAATTGAGGCCAACAG
Cpt1a	NM_031559.2	GCAATAGGTCCCCACTCAAG	ATGATGCCATTCTTGAACCG
Cpt2	NM_012930.1	GAATTTTGAGACTGGCGTTG	CTTTTGGAAGGGTTCAAGTG
Acaa2	NM_130433.1	AGGATTAACGGATCAACACG	CTTGGTCTTCACCTCAATGG
Gpat	NM_017274.1	CCACATCAAGGATACAGCTC	CGTGCATGAATAGCAACACC
Dgat2	NM_001012345.1	TACCTACCTCGGATCTCGACC	CGGAGTAGGCAGCGATGAG
Mttp	NM_001107727.1	AGTGAACCCGTGACAGAACC	TTGGAACCTTCCCAGCTACG
Tnfa	NM_012675.3	ACTACGATGCTCAGAAACAC	AATAGAGGGGGTTCCGTAAG
IL6	NM_012589.2	GACTTCACAGAGGATACCAC	ACAAACTCCAGGTAGAAACG
IL1b	NM_031512.2	TGTGGATCCCAAACAATACC	ATAGTGCAGCCATCTTTAGG
IL10	NM_012854.2	TCCCCTGTGAGAATAAAAGC	ATGTCAAACTCATTCATGGC
Cxcl1	NM_030845.1	TCGATGGTCGTTCAATTCC	ATACAACATAGCCTCTCACAC
Cxcl2	NM_053647.1	TCGTAATGTGAATATCCCCTG	CCCCACTTCAAGACATGAG
Tgfb	NM_021578.2	GCAACAACGCAATCTATGAC	CCACAGTTGACTTGAATCTC
Col1a1	NM_053304.1	AACCAAAAGTGCATTCAACC	GGCAGAAAGGGACTTATACC
Ccn2	NM_022266.2	AACTGATAGCCTCAAACTCC	TCTGACTTCTGATCCATTGC
Acta2	NM_031004.2	TGAAGTATCCGATAGAACACG	AGCACAATACCAGTTGTACG
Srebp2	NM_001033694.1	GCCGAACTGGGCGATGGATG	ACCTGGCTGAATGACCGCTG
Sqe	NM_017136.2	GGCTCAGGCGTTGTATGAAC	GCAACGGAGAAGAAGTGTCG
Hmgcr	NM_013134.2	CCTCCATTGAGATCCGGAGG	GTGAGGATGATGATGTCGCTG
ubc	NM_017314.1	CGTACCTTTCTCACCACAGT	ATTCAAAGTGCAATGAAACTTGT
b2m	NM_012512.2	TGTGGCTGGAGGTTTAGTCC	AACAGAAGGGCAGAAGACGC
gapdh	NM_017008	CTTGTGCAGTGCCAGCCTC	TCCCGTTGATGACCAGCTTC
